# Pre-Concentration and Analysis of Mycotoxins in Food Samples by Capillary Electrophoresis

**DOI:** 10.3390/molecules25153441

**Published:** 2020-07-29

**Authors:** Raffaella Colombo, Adele Papetti

**Affiliations:** Department of Drug Sciences, University of Pavia, Viale Taramelli 12, 27100 Pavia, Italy; raffaella.colombo@unipv.it

**Keywords:** food quality, food safety, health, mycotoxins, contaminants, residue analysis, capillary electrophoresis, pre-concentration techniques, microchip-CE

## Abstract

Mycotoxins are considered one of the most dangerous agricultural and food contaminants. They are toxic and the development of rapid and sensitive analytical methods to detect and quantify them is a very important issue in the context of food safety and animal/human health. The need to detect mycotoxins at trace levels and to simultaneously analyze many different mycotoxin types became mandatory to protect public health. In fact, European Commission regulations specified both their limits in foodstuffs and official sample preparation protocols in addition to analytical methods to verify their presence. Capillary Electrophoresis (CE) includes different separation modes, allowing many versatile applications in food analysis and safety. In the context of mycotoxins, recent advances to improve CE sensitivity, particularly pre-concentration techniques or miniaturized systems, deserve remarkable attention, as they provide an interesting approach in the analysis of such contaminants in complex food matrices. This review summarizes the applications of CE combined with different pre-concentration approaches, which have been proposed in the literature (mainly) in the last ten years. A section is also dedicated to recent microchip–CE devices since they represent the most promising CE mode for this application.

## 1. Introduction

Mycotoxins are natural secondary metabolites produced by filamentous fungi (*Aspergillus, Fusarium,* and *Penicillium*). They mainly include aflatoxins (AFs), trichothecenes, fumonisins, ochratoxin A (OTA), patulin (PAT) and zearalenone (ZEA) [1,2,3]. Nowadays, about 500 mycotoxins are currently known, and they contaminate almost 40% of cereals globally produced [4].

In addition to the known mycotoxins, there are also the so called “emerging mycotoxins”, which refer to those fungal metabolites that could be toxic, which we have no control of and or restrictions against [5,6]. Non-regulated mycotoxins constitute a very important issue as their toxicity is not known [6]. The number of these mycotoxins is very alarming, as confirmed by a recent study on different maize samples from Serbia, which pointed out the presence of more than one hundred non-regulated fungal metabolites in samples cultivated over four years [6,7].

Mycotoxins represent dangerous contaminants of agricultural products and foodstuffs (mainly cereal products, fruits, vegetables, and derived products, such as wine and juices) and toxicological agents for animal and human health [3,8]. Their riskiness is related to their high stability over time, also at high temperatures [3]. Their toxicity concerns teratogenic and/or mutagenic effects, and some of them (aflatoxin B1-AFB1, aflatoxin M1-AFM1, OTA) are human carcinogens [1,2,4,9]. Others, such as ZEA, exhibit carcinogenic, hematotoxic, and hepatotoxic properties only in in vitro experiments and in some animal species [2,3,9]. They can also cause immunosuppression and hormonal disorders [2,9]. In addition, the few works on mycotoxins in pet food showed their high toxicity in relation to pets’ health [10].

The main issue regarding human toxicity is correlated to animal toxicity. In fact, AFs, OTA, ZEA, ergot alkaloids, and fumonisins were particularly toxic for farm animals, mainly non-ruminants and cereal grains or forages remain the principal food source which can affect animal and human health [9].

Considering their abundance and toxicity, a careful evaluation of risk assessments has caused the European Commission (EC) to adopt restricted regulations to control their limits in foodstuffs [1,11,12,13] with specific official methods [12]. The control of food and feed is fundamental during production and storage phases [4]. Standardized methods for determination of different mycotoxins in many food matrices mainly consist of Thin-Layer Chromatography (TLC) and High-Performance Liquid Chromatography (HPLC) [14].

The methods must guarantee the determination and quantification of trace levels of mycotoxins in complex food matrices and biological samples, as mycotoxins are mainly produced in a very low concentration [1]. Another great challenge in mycotoxin analysis is related to the fact that a single filamentous fungus can produce many different mycotoxins, so it is necessary to have rapid multi-analyte methods [15]. This became mandatory as human health could be at risk when simultaneously introducing more than one potential source of mycotoxin in the diet. The immunoaffinity methods, which use very selective immobilized antibodies, do not allow the analysis of multi-mycotoxins, but they are a part of the clean-up procedure and are used in a multi-approach involving a careful setup of sample preparation (extraction/clean-up) prior to analysis [16].

Gas Chromatography-Mass Spectrometry (GC-MS) and, mainly, HPLC-UV or HPLC-MS represent the most applied and sensitive analytical techniques to detect and quantify different mycotoxins and their metabolites in different samples, such as, for example, cereals, beer, and dairy products [7,17,18,19,20,21,22]. A sample pre-concentration step by using mainly solid phase extraction (SPE) [14,18,23,24] and immunoaffinity columns (IACs) [14,21,23,25,26,27] is always fundamental, even if such sensitive analytical techniques are generally used to detect trace levels. Particular sorbents, such as aptamers, are often used as materials for SPE or microchips [23]. Liquid-Liquid Extraction (LLE) [28], Pressure Liquid Extraction (PLE) [29] and Dispersive Liquid-Liquid Microextraction (DLLME) [30,31] are less common and therefore only few recent publications discuss their use.

In Capillary Electrophoresis (CE) the application of voltage allows a short and efficient analysis, and the free-solution environment with only small percentages of organic solvents, together with the small consumption of samples and buffers, ensures economic benefits and environmental compatibility [32,33,34]. In the last 30 years, CE and its different modes (e.g., Capillary Zone Electrophoresis (CZE), Capillary Electrochromatography (CEC), Nonaqueous CE (NACE) or Micellar Electrokinetic Chromatography (MEKC)) have found many applications in food safety controls [35,36]. 

Many CE parameters, such as background electrolytes (BGE), in terms of pH, type and concentration, capillary type and length, capillary conditioning (type and time), voltage, injection mode and parameters, as well as temperature, can be set up, making CE a versatile analytical technique. In addition, each CE mode exploits a simple modification of CZE BGE (by adding organic solvents for CEC and NACE or surfactants for MEKC) and different basic principles (mobility, hydrophobic/ionic interactions, etc.) [35,36]. This ensures a wide range of applications in the analysis of food-related molecules: amino acids, lipids, carbohydrates, DNA, vitamins, and different contaminants (metals, pesticides, herbicides, fungicides, drugs, and mycotoxins) [35,36]. CE theoretical principles and different CE modes could represent the right key to separate mycotoxins, which have a very different chemistry [17]. In addition, MEKC, exploiting the CZE basic principle of mobility and the HPLC theory of interaction, could allow us to increase the versatility of CE applications [36].

Polymerase Chain Reaction (PCR) protocols represent important routine techniques for the detection of potentially mycotoxigenic molds to prevent the mycotoxin presence in food matrices [37] and are associated with sensitive methods, such as HPLC-MS, CE, and particularly MEKC [38,39]. In addition, new offline and online/in-capillary pre-concentration techniques (in sample preparation step) or the use of sensitive detectors such as MS [36,40] and Laser-Induced Fluorescence (LIF) allowed food contaminants and residue analysis by CE [36,40,41,42]. Microchip-CE devices are mainly proposed when rapid analyses are needed to point out frauds and contaminations [36,43,44,45].

In the last years, few reviews were published on this topic [8,43], and previous works were focused on suggested approaches in sample preparation [46] or CE detection systems (LIF) [47], exploiting the native fluorescence of some mycotoxins (AFs, OTA and ZEN) or the possibility to derivatize them with cyclodextrins (CDs) [47].

**Figure 1:**
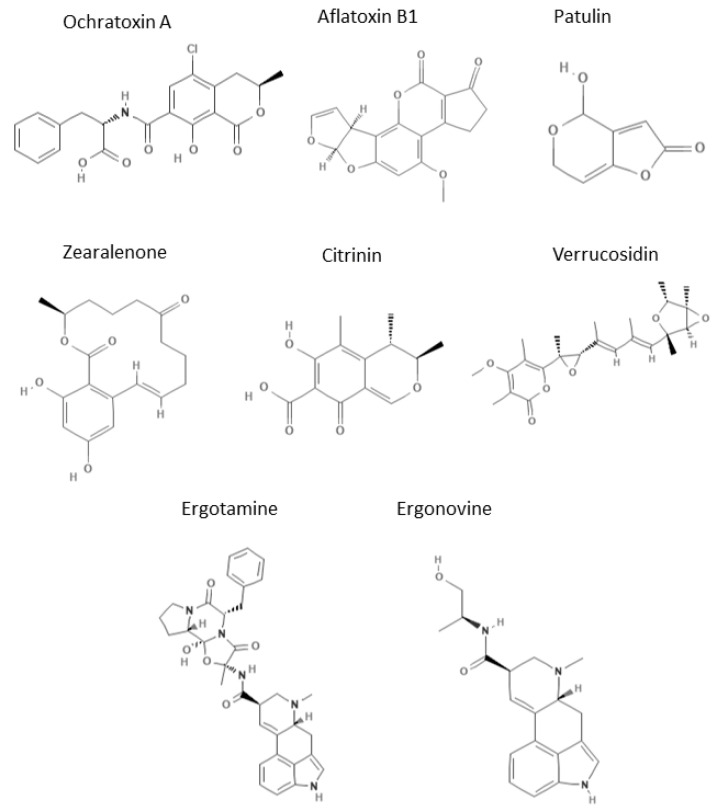
Chemical structures of mycotoxins analyzed by Capillary Electrophoresis (CE) techniques.

The aim of this review is to critically discuss CE modes in mycotoxin analysis, mainly focusing our attention on the trends and advances of recent decades, which allowed researchers to improve CE limits, developing methods able to rapidly carry out trace analysis in complex food matrices. In fact, nowadays, recent advances in CE techniques ensure the same sensitivity levels as HPLC methods, allowing the application of this technique for routine monitoring. Despite these improvements, chromatography still represents the elective technique to analyze mycotoxins, but a CE overview of its recent advances in this topic is necessary to underline CE potentialities. This review will be subdivided into two main sections concerning different pre-analysis approaches for sample preparation and the most recent CE analysis strategy in mycotoxin detection (i.e., microchip-CE). Figure 1 reports the main mycotoxins analyzed by CE techniques, which are discussed in the review.

## 2. Sample Preparation for CE Analysis

### 2.1. SPE Procedures

Nowadays, offline and online SPE methodologies have completely replaced LLE techniques in food analysis thanks to their selectivity, lower time and solvent consumption, and the great number of SPE variants, such as dispersive SPE (d-SPE), Magnetic SPE (MSPE), Stir Bar Sorptive Extraction (SBSE), Solid-Phase Microextraction (SPME), and Micro-Solid-Phase Extraction (μ-SPE) [48,49,50,51]. 

SPME was a very promising technique, as it was able to integrate sampling, sample preparation (extraction), concentration, and sample introduction into the capillary in a single step without the need for sample transfer. Its advantages are evident in food analysis as the food matrix is very complex, containing compounds (inorganic and organic substances) with different physico-chemical properties and at different concentrations (often also in traces). In addition, SPME is also suitable for low-volume applications [52,53]. Developments in automation, such as new autosamplers, and high-throughput analyses of fibre-SPME setups allowed researchers to increase the speed of sample preparation and injection procedures, allowing them to be applied in routine analysis [52].

In all these approaches, the research on Molecularly Imprinted Polymers (MIPs) was fundamental. MIPs are stable polymeric sorbent materials with a high specificity, which are very useful in pre-concentration steps of food and environmental analytes. In addition, MIPs are also able to reduce matrix interference, thus increasing method sensitivity [49]. The SPME procedure coupled with MIPs is named Molecularly Imprinted Solid-Phase Microextraction (MISPME) and represents a very promising and versatile technique, whose different formats and coatings ensured a wide range of applications, including food analysis [54].

#### 2.1.1. Offline SPE

**Figure 2:**
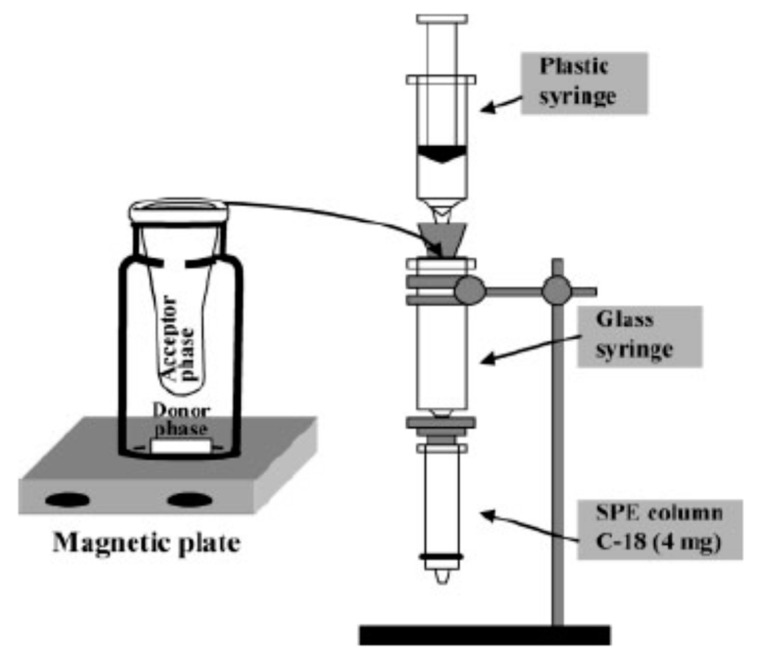
Equipment of SLM device set-up by Almeda et al. to extract and preconcentrate ochratoxin A (OTA) from wine samples. (Reproduced with permission from Almeda, *Electrophoresis*; published by Wiley-WCH, 2008 [56]).

SPE has been widely used to analyze O0TA, which is a very stable mycotoxin present in different food matrices, mainly cereals and wine, but also dried fruits, coffee, and cocoa [51]. OTA is nephrotoxic, teratogenic, carcinogenic, and immuno-suppressive in many animal species [2], and represents a high risk for human health, as it is classified as a possible human carcinogen in group 2B, as AFM1 [2,55]. Its maximum permitted level (0.5–80 μg/Kg) in different foodstuffs has been established by the EC [12,13]. The study of Almeda et al. represented a turning point in the analysis of OTA, as it was the first example of offline SPE combined with CE to detect this mycotoxin by CZE-UV in wine samples [56]. OTA is a commonly present in wine at low levels (<1 μg/L), and an efficient pre-concentration step is fundamental. This study proposed an interesting approach, thanks to the application of a micromembrane extraction with a supported liquid membrane (SLM) device combined with SPE. In this device, wine is the donor phase (pH 8), while water is the acceptor one (pH 11) positioned in a micromembrane (Figure 2). To obtain the best performance with SLM devices some parameters have to be optimized: the organic solvent used to extract the analyte (OTA is a low-polar substance), the diffusion mode of the analyte, stirring conditions with a magnetic plate and bar, and the volume and pH of the two phases. This combination made possible the study of mycotoxins by CZE-UV, by simply using a neutral pH BGE with a small addition of βCD, eliminating matrix interference and offering a rapid and simple clean up with high selectivity. The pre-concentration procedure allowed sensitivity values higher (30 μg/Kg) than those obtained with the most recent optimizations of SLM procedures, combined with different analytical approaches, such as Surface-Enhanced Raman Spectroscopy (SERS) or HPLC-MS [57].

#### 2.1.2. Online and Inline SPE

Until 10 years ago, offline SPE approaches were very diffused because of their simplicity, but they had some limits in terms of simultaneous determinations and high-throughput analyses [58]. For this reason, online SPE procedures have been optimized and are currently widely used in the preparation of mycotoxin samples as they improve the method sensitivity, are less time consuming, and they also improve the throughput. The literature is rich with works which propose online/inline SPE procedures and LC-MS techniques in the study of different mycotoxins (i.e., AFs, ochratoxins) especially in dairy products and wine, obtaining rapid and sensitive analyses [59,60,61,62]. Regarding CE, online procedures can make this technique competitive with LC methods. In fact, in online systems SPE columns are connected with CE, while, in inline procedures, SPE is integrated with the CE capillary, that is, the SPE sorbent material is positioned along the CE capillary. Inline SPE allows for minimal sample loss, as the sample enrichment occurs directly in the capillary [41]. Over the years, many SPE different formats, configurations, and sorbent materials have been developed to perform very sensitive trace analyses of complex biological, environmental, and food samples [63]. Almeda et al. continued their studies on mycotoxin analysis with the optimization of SPE procedures, considering OTA as a model mycotoxin and obtaining extraction methods with an inline SPE sensitivity one order of magnitude higher than that of offline SPE [58].

The micro SPE (μSPE) procedure works with SPE principles, but the extraction, clean up, and pre-concentration occur in a single step. μSPE is widely used in the analysis of complex food matrices thanks to the capability of the sorbent, which is packed inside of a porous polymer membrane, to clean very dirty samples [50]. 

**Figure 3:**
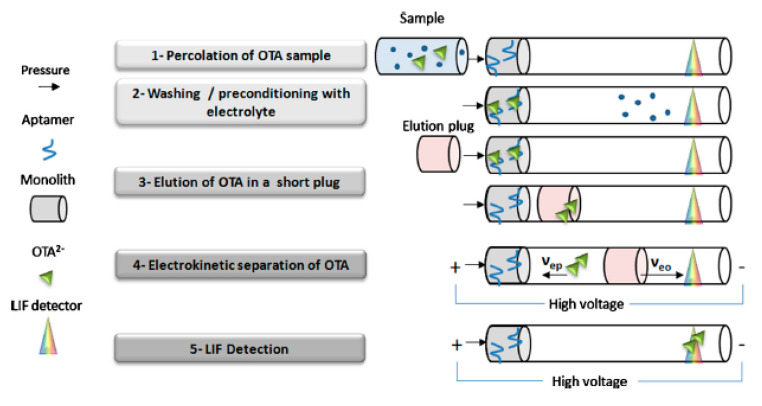
Inline aptamer affinity CE in OTA analysis. In the image, the complete procedure is described. 1. Percolation step: OTA(-cations species) is solubilized in binding buffer and percolated through the column with aptamers; 2. Washing: OTA is bound to aptamers, while unbound analytes and binding buffer are flushed away by the separation buffer (the presence of ions maintain the complex OTA-aptamers); 3. Elution: OTA is desorbed by injecting an elution plug (plug length must be >than 10% the monolith length); 4. Electrokinetic separation: OTA is separated by voltage. 5. Detection: OTA reaches Laser-Induced Fluorescence (LIF). (Reproduced with permission from Marechal, *Journal of Chromatography A*; published by Elsevier, 2015 [41]).

An interesting online μSPE-CE system based on aptamers and not on antibodies was successfully used to improve the enrichment step. Aptamers are single-stranded DNA or RNA molecules with a high and stable binding capacity for different type of molecules, from drugs to proteins and cells. Many examples of OTA-binding aptamers are present in the literature, showing high selectivity and recovery (extractions in the order of μg/Kg) [23]. This type of inline pre-concentration step using a vinyl-silanized monolith capillary filled by an aptamer solution, integrated with CZE-LIF, allowed researchers to quantify OTA up to the ppb level (limit of quantification-LOQ: 0.1 pg) in wine and beer samples (Figure 3) [41]. 

An aptamer probe with a vinyl-silanized monolith capillary as affinity ligand was used in aptamer affinity CE. An interesting approach in mycotoxins analysis consisted of Metal Cation Mediated free zone CE (MCM-CE) in which aptamers with different sizes exhibited different mobilities because of their different interactions with cations (Mg^2+^ or Ca^2+^) [41]. The high binding affinity and stability of aptamers combined with high sensitivity techniques represent an interesting strategy in mycotoxin analysis. 

An aptamer CZE-LIF method was set up for the analysis of AFB1 in corn flour samples, obtaining high sensitivity values (LOQ: 0.5 nM). AFB1 was classified as a Group 1 carcinogen [55] and, in addition to hepatocellular carcinoma, or primary liver-cell cancer, it could also be responsible for respiratory cancer in animal models and humans. It is the most potent carcinogen known in foods [1,2]. A fluorescent aptamer and a complementary DNA (cDNA) were used to form a duplex DNA, whose dissociation is caused by the presence of AFB1. The presence of cations is fundamental to obtain a good separation of fluorescent aptamers and duplex DNA. Parameters such as the length of cDNA, concentrations of aptamers and cDNA, and their ratio must be carefully optimized together with the composition of BGE and the concentration of MgCl_2_ added to the sample and run buffer (BGE) [42].

### 2.2. Immunoaffinity Capillary Columns (IACs)

Immunoaffinity capillary columns are filled with antibodies, and in contrast to SPE columns, the analyte is retained and the interferents are eluted [16]. Notwithstanding time and costs, in general, immunoaffinity extraction has a high binding affinity and selectivity for mycotoxins as OTA and AFs and therefore it represents one of the most used pre-concentration approaches [16,23]. Immunoaffinity capillary columns (IACs) represent a robust and sensitive (ng/kg) method to purify AFM1 from dairy products with high recovery and precision. The only disadvantage consists of higher costs than SPE, but the advantage of detecting AFM1 in milk and dairy products overrides this. In fact, this aflatoxin is related to the presence of AFB1 in animal feed. AFM1 is hepatotoxic and carcinogenic and represents a high risk for human health as dairy products are highly consumed in the daily diet and also because of its stability in milk processing and storage [16].

Regarding inline IACs, Chamieh et al. set up a combined system in which a micro-immunoaffinity column (μIAC), based on an acrylate monolithic support, was coupled inline with a CE-coated capillary to analyze OTA. The careful control of the reproducibility of the capillary monolith preparation ensured a high and constant μIAC binding capacity, allowing the authors to obtain a robust method without a high consumption of the sample and solvents, which occurred in offline procedures [27]. An IAC-CZE-UV method was applied to the detection of citrinin (CIT), a nephrotoxic and hepatotoxic mycotoxin diffused in the Balkans and Asia [64] in red yeast rice and Monascus color. This method could simplify the laborious sample preparation required by HPLC, but particular attention to the choice of eluting solution conditions (solvent concentration, type, and pH) has to be paid as the coupling efficiency (%) closely depends on them. A modified silica column was activated and covalently bound with an anti-CIT antibody to obtain good recovery at levels of μg/kg [64].

### 2.3. Sweeping Techniques

Sweeping represents an online concentration procedure used to increase CE sensitivity from 10- to 5000-fold. In sweeping, a neutral or charged pseudostationary phase (mainly polymers or micelles) is added to BGE. The mechanism of pre-concentration is based on the partitioning or interaction of the analyte with the pseudostationary phase, and it is due to the application of CE voltage at negative polarity, by using MEKC mode for separation [65,66]. Sweeping is often combined with a previous sample stacking obtained by an electrokinetic injection (field-enhanced sample injection (FESI)), optimizing the conductivity of the sample buffer and BGE. Therefore, sample stacking and sweeping are two different online concentration procedures, and their combination, which is known as cation-selective exhaustive injection and sweeping (CSEI-sweep), can further improve sensitivity [65].

The first example of sweeping approach-MEKC in mycotoxin analysis dates back to 2010. In this work, an IAC and a sweeping procedure were combined with MEKC-LIF, allowing the detection and quantification of AFB1, AFB2, AFG1, and AFG2 in rice samples. The sweeping-MEKC pre-concentration step has been carefully optimized in terms of sample solvent and injection time, allowing a sensitivity (LOQ range values: 0.13–1.74 μg/L) comparable to that obtained by HPLC-Fluorescence Detector (FD) [67]. 

### 2.4. Liquid–Liquid Extraction (LLE) 

LLE is based on a distribution of an analyte between two immiscible solvents (aqueous and organic phases). The equipment is simple and the procedure is low cost, but its application has been complicated by a critical point, that is, the choice of the solvents, which must consider a lot of parameters: partition coefficient, density, viscosity, interfacial tension, volatility, selectivity, and stability [68].

Few LLE variants were applied to mycotoxin analysis, and were mainly used with chromatographic approaches [28,29,30,31]. In fact, as mentioned above, nowadays, the LLE approach has been completely overcome by SPE procedures in mycotoxin analysis, mainly because of LLE’s low efficiency and long extraction times. In addition, for example, for some carboxylic mycotoxins, LLE efficiency was low and also extracted residues [46].

**Figure 4:**
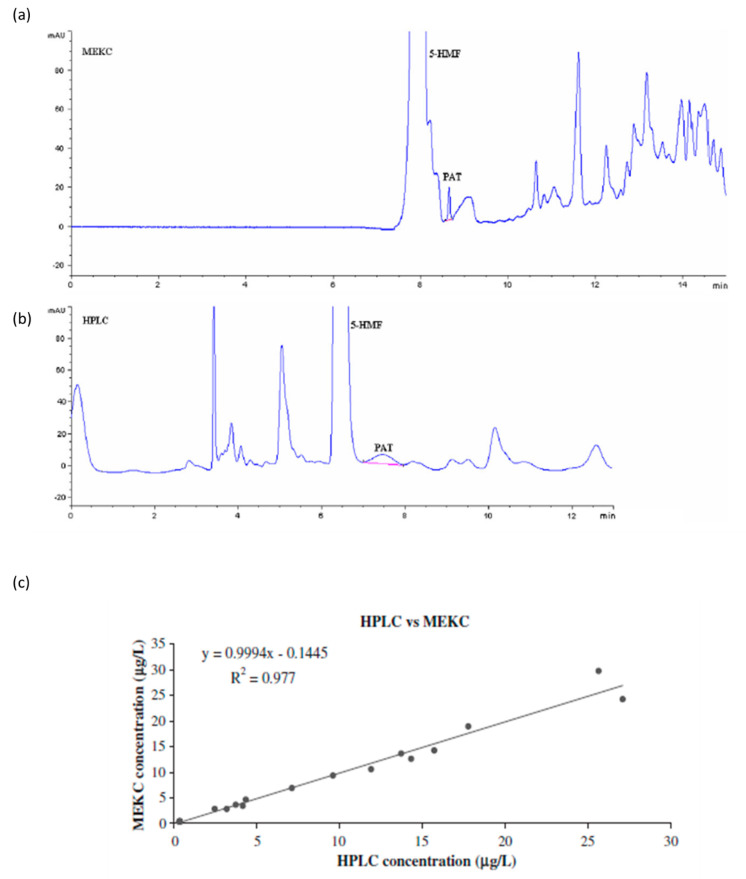
Patulin (PAT) analysis in apple juice by Liquid-Liquid Extraction (LLE) combined with (**a**) Micellar Electrokinetic Chromatography (MEKC)-UV and (**b**) HPLC-UV. (**c**) Plot of MEKC and HPLC concentrations. The results obtained by the two methods were strictly correlated. (Reproduced with permission from Murillo-Arbizou, *Food and Chemical Toxicology*; published by Elsevier, 2010 [69]).

Notwithstanding this, a simple LLE approach, used by Murillo-Arbizou et al. to extract PAT from apple juices for infants, can be cited. PAT is mainly present in fruits and it is relevant to the need for methods for the analysis of apple-based infant foods in relation to their high and common consumption. This mycotoxin has a cyclic γ-lactone structure. Ethyl acetate and acidified water were used as solubilizing solvents in a sample preparation step before MEKC-UV analysis. The obtained results were compared to those obtained by a previously validated HPLC-UV method, confirming a good potentiality for the new MEKC method proposed here (Figure 4) [69]. The same sample preparation procedure was also coupled with a CZE method, once again in the analysis of PAT in apple juices, with a sensitivity very similar to that of the HPLC method [70]. 

Vortex-Assisted Low Density Solvent Microextraction (VALDS-ME) is a very recent evolution of the DLLME technique. VALDS-ME consists of the use of non-toxic and low-density extraction solvents, followed by a vortex agitation and, in the final step, by the addition of a salt demulsifier to induce the separation and act as a substitute for centrifugation. It was set up for the first time to analyze AFs in rice samples by HPLC-FD [71] and then this approach was applied in combination with CZE-LIF to detect AFs (AFB1, AFB2, AFG1, and AFG2) in agricultural products [72]. The method of Gao et al. was able to rapidly separate (in about 6 min) AFs with high efficiency and sensitivity (LOQ range 7–300 ng/L) in food and dairy cattle feed samples, with results completely comparable to those obtained by HPLC-FD and UHPLC-MS/MS [72].

The use of supercritical fluids and, in consequence, Supercritical Fluid Extraction (SFE) was also explored in combination with CE. ZEA is a non-steroidal estrogenic mycotoxin, which is not classifiable (Group 3) for its carcinogenicity in humans [55], but it was recognized to have carcinogenic properties in in vitro experiments and in animals [2,3,9] and to cause changes in female reproductive organs with potential implications for infertility and cervical cancer [3,73]. ZEA and its metabolites (α-zearalenol (α-ZOL), and β-zearalenol (β-ZOL)) were analyzed by CZE-Amperometric Detector (AD) after a SFE step by Arribas et al. [74]. Supercritical carbon dioxide and methanol were used in a polar cartridge made of magnesium oxide and silica gel to efficiently clean up mycotoxins from maize flour samples. The careful optimization of the methanol percentage together with the voltage value allowed for a good resolution in the three analytes. In addition, the polyphenolic moieties of ZEA, α-ZOL and β-ZOL, together with a 14-membered lactone cycle (only differing for the substituent located at the 6’-position) are subjected to an electrochemical oxidation to allow for the use of AD, thus obtaining a good sensitivity (20–35 μg/L) [74].

### 2.5. PCR Protocols

Real-time quantitative PCR (RTi-PCR) is well known to quantify DNA, but it can be also used to analyze mycotoxins, such as PAT. The critical point consists of the need to optimize sensitive protocols in order to have PAT-producing and non-producing strains. Fluorescently labeled oligoprobes, such as TaqMan probes, are particularly promising thanks to their selectivity, accuracy, and sensitivity [38].

A similar approach has been also applied to detect verrucosidin in dry-ripened (cheese), dry-fermented (sausages), and dry-cured (ham slices) foods. Verrucosidin is a tremorgenic mycotoxin associated with neurological disorders [39]. Duplex real-time PCR (qPCR) with a TaqMan probe has been developed and combined with MEKC-UV or HPLC-MS, obtaining similar results. qPCR detects molds which can produce secondary metabolites, generating mutant fungal strains potentially involved in mycotoxin production. To quantify the molds involved in the production of mycotoxins would allow us to prevent the risk of their formation. In the method of Rodríguez et al., an internal amplification control was used in the optimization of PCR, allowing a higher sensitivity due to the elimination of interference and to a rapid quantitative determination of verrucosidin-producing molds [39]. This method monitoring the quantification of produced molds was proposed as an approach to control and prevent the formation of verrucosidin in drying and ripening food processes.

### 2.6. Cloud Point Extraction (CPE) 

Cloud Point Extraction (CPE), also named surfactant CPE, is based on the use of small amount of non-ionic and anionic surfactants over their critical micellar concentration (CMC), which selectively solubilize inorganic or organic analytes [75]. The critical point of this procedure is the choice of type and concentration of surfactant in relation to analyte parameters, such as structure, hydrophobicity, capacity to create hydrogen-bonds, molar refraction, and dipolarity [75]. Moreover, the presence of salts/additives must be controlled to optimize CPE performance, as they can modify the cloud point temperature (CPT) of surfactant [75,76,77]. CPE consists of a micelle-extraction procedure, in which the separation occurs in the micellar hydrophobic core. The advantages are efficiency, low cost and rapidity thanks to the combination of extraction and pre-concentration in a single step [75]. In addition, CPE represents a green approach particularly suitable for food processing [76]. In fact, it is commonly used for the extraction of bioactive compounds in foods, as well as proteins [76], and also metal ions [76,77]. CPE works at a mild-low temperature; non-ionic surfactants form clouds until they reach the temperature (CPT) at which phase separation occurs, thus promoting the formation of two phases, the first one rich in surfactants and concentrated analytes, and the second saturated and containing surfactants [76,78]. These conditions are particularly appropriate for food molecules sensitive to temperature [76]. 

Only a few papers about CPE combined with CE are present in the literature, and they refer to CZE or MEKC modes. The CZE limit is linked to the potential absorption of surfactants in the capillary wall with aqueous BGE, and this can cause irreproducible results. This disadvantage can be overcome by using non-aqueous BGE (acetonitrile-methanol), as in NACE modes, or by adding surfactants (MEKC) or additives to the BGE [75]. CPE can be used in mycotoxin analysis in food matrices, exploiting the formation of hydrophobic interactions and hydrogen bonds useful for mycotoxin extraction [76].

**Figure 5:**
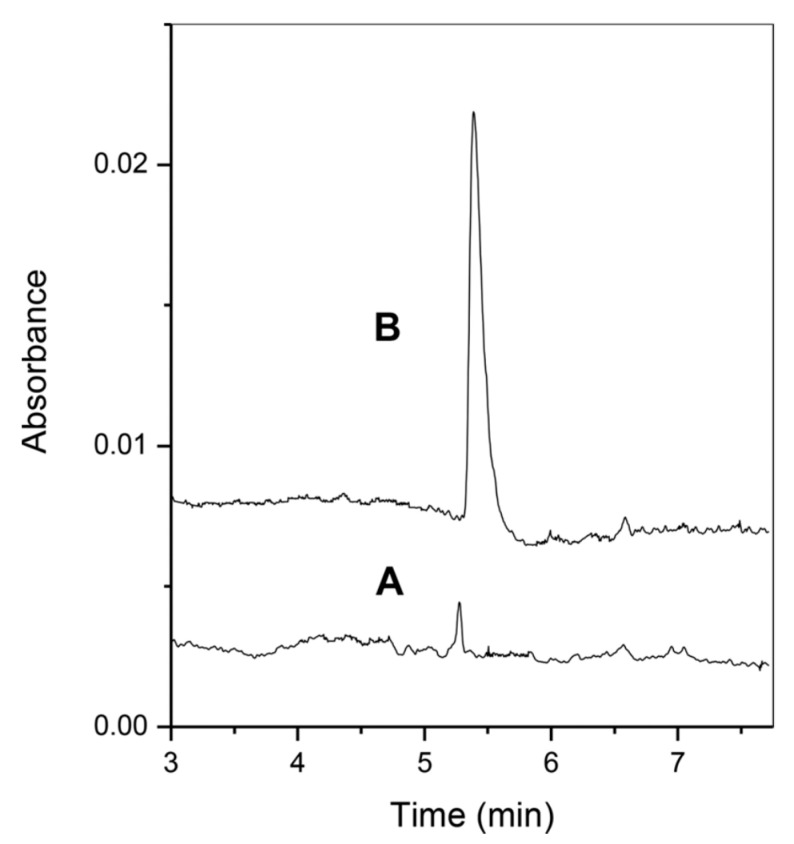
Comparison of ergot alkaloids analysis in rye flour sample before (**A**) and after (**B**) CPE procedure combined with CZE-UV. (Reproduced with permission from Felici, *Electrophoresis*; published by Wiley, 2015 [78]).

Mycotoxins such as ergot alkaloids (ergotamine and ergonovine) were analyzed in cereal samples combining CPE and CZE-UV, whose sensitivity was improved in comparison to CZE alone. In fact, a pre-concentration factor of 22 was obtained (Figure 5). The critical step consisted of the optimization of surfactant concentration, extraction buffer pH, and centrifugation time to accelerate phase separation [78].

In Table 1, the pre-concentration procedures and CE modes according to mycotoxin type are reported. 

## 3. CE Analysis

### Microchip-CE

Microfluidic technology resulted in many advantages such as a reduction in sample and reagent consumption, rapid analysis and high sensitivity. Microchip devices include sample pretreatment, solution distribution/mixing, separation, and detection [79]. The optimization of different format and material expanded microchip applications from clinical to food and environmental analysis [36,45,79].

Microfluidic technology combines different steps: sample preparation (filtration, enrichment), analytes separation, and detection. This approach is very useful to solve the problem of exposure to trace contaminants such as mycotoxins [43,80]. 

A lot of interesting microchip-CE devices were set up and proposed for mycotoxin analysis in different matrices around the 2000s [43]. These devices are also named CE microfluidic immunosensors and are particularly useful to detect food frauds and contaminants thanks to their rapidity, sensitivity, and reproducibility. In addition, these systems reduce costs, allowing sample preparation on the same device and consuming only small volumes of solvents and samples [36]. The design, fabrication method, and materials of microfluidic immunosensors represent the key points of these devices [43]. Glass and silicon are the most used materials, often in combination with polymers, which allow different fabrication methods and a broad range of applications. The fluidic system can be controlled by pumps (hydrodynamic approach) or by electrodes (electrokinetic approach) and formats can differ for flow types: flow through, also named electromigration chip, or lateral flow test strips (LFTS) [43,81]. LFTS represented a tricky approach because of their strong matrix dependence and, in consequence, their poor performance in terms of selectivity and sensitivity [43]. The step of the immunoreagent immobilization is fundamental and different immobilization procedures and supports affect the fluidic system and device performance [81]. In mycotoxin analysis, optical electrochemical (ECL) detection and, often, amplification techniques are used to obtain a good sensitivity [43]. Today, microchip-CE certainly represents the best CE strategy in mycotoxin analysis.

A promising microchip-CE-ECL was proposed by Hervás et al. for ZEA detection in infant cereal milkshakes. A double-T glass microchip, made of a support of magnetic beads, was designed with two separated channels for immunological and enzymatic reactions, and this solves the common problem of non-specific adsorption between protein and wall channels, allowing a limit of detection-LOD value of 0.4 μg/L, good precision, and accuracy [81]. The microchip-CE technique applied to ZEA analysis has the advantage of obtaining a higher sensitivity (two orders of magnitude) than CZE-LIF methods which must be set-up with the addition of CDs to increase fluorescence and method sensitivity [82]. In fact, in the past, CZE-LIF with CDs has been widely used for AFs, OTA and ZEA [47,82].

Some chips are projected with covalent conjugated immobilizations, which allow for rapid chip regeneration and reproducible data for 20 analyses. For example, a regenerable glass microchip with a peptide-OTA conjugate has been set up to detect OTA in green coffee extracts. A chemiluminescence detection has allowed the conjugate reach a good LOD (0.02 μg/L) even if the matrix effect compromised the chip performance and potentiality for OTA screening [83]. This array immunosensor represents an important starting point in OTA analysis by microchip-CE and, in recent years, many authors developed different sensors, based mainly on ECL detection, such as MIP-ECL sensors [84]. This sensor has similar sensitivity to that previously obtained by Sauceda-Friebe et al. or enzyme-linked electrochemical immunosensors, and is able to reach high-performance LOD values (0.008 ppb), even if these values are lower than those established by the EU Regulation Commission [85]. 

In the analysis of OTA, the systematic evolution of ligands by the exponential enrichment (SELEX) procedure can also solve the problems with the limits related to enzyme-linked electrochemical and array immunosensors, such as the matrix effect, false positives, and negative results [23]. It consists of the use of aptamers, and these aptasensors exhibited a high specificity for OTA and, combined with fluorescence detection, they obtained good sensitivity values [86].

**Figure 6:**
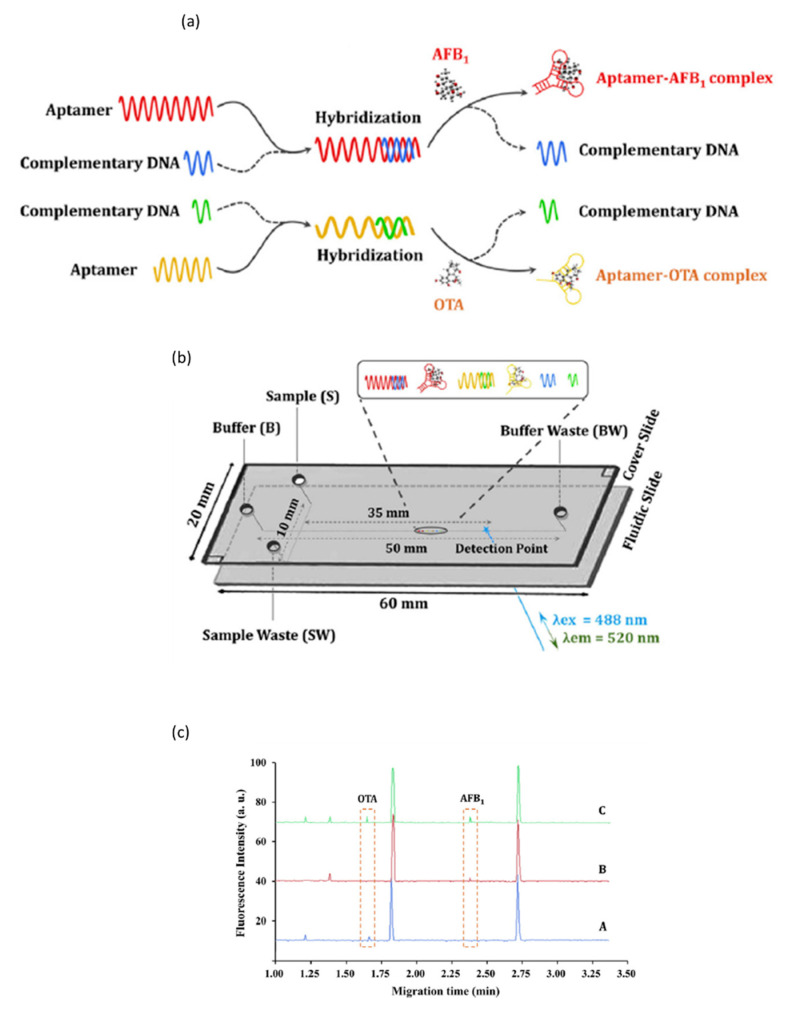
Aptamer-based microchip-CE-LIF in the simultaneous analysis of AFB1 and OTA. (**a**) complex formation procedure; (**b**) microchip platform; (**c**) electropherograms of (**A**) OTA in presence of aptamer-AFB1/C-aptamer; (**B**) AFB1 in presence of aptamer-OTA/C-aptamer and (**C**) simultaneous analysis. (Reproduced with permission from Xiao, *Journal of Chromatography A*; published by Elsevier, 2018 [44]).

An interesting application of an aptamer-based microchip-CE was proposed by Xiao et al. for the simultaneous determination of AFB1 and OTA. A C-aptamer, which is the partially complementary DNA of aptamers, was used. This setup exploited the different lengths of aptamers, which are able to simultaneously detect different analytes with high specificity and resolution when combined with LIF detection [44]. The aptamer was made fluorescent by using Synergy Brands, Inc (SYBR^®^) gold, whose different dilution times in buffer were carefully investigated to optimize the analyte migration time and peak intensity (Figure 6a,b). The analysis was very rapid (3 min), obtaining LOD values around 0.0020 ng/mL, which are suitable for trace analysis in foodstuffs as cereals and different types of oil Figure 6c [44]. Table 2 summarizes the main microchip-CE applications in mycotoxin analysis.

## 4. CE Potentialities and Future Perspectives

Considering CE principles and the capacity of the methods present in the literature, it is important to contrast the low CE diffusion, providing interesting future perspectives for this technique in mycotoxin analysis. The methods proposed here obtained sensitivity values in complete agreement with the limits fixed by the EC (Table 3). Below, the CE key factors and strategies studied in the works cited in this review are summarized, focusing our attention primarily on the main mycotoxins analyzed by CE techniques (OTA and AFs).

- Analyte solubility: Mycotoxin chemical structures are different, as is their water solubility, ranging from the water-soluble PAT to the water-insoluble CIT, with most mycotoxins (OTA, AFs, ZEA, verrucosidin, and ergot alkaloids) possessing a low water solubility. It is well known that the first parameter for which CE can be chosen is generally the water solubility/affinity of the analyte, as CE works in the presence of an aqueous buffer in the so-called free-solution approach. Considering OTA and AFs, OTA is a phenylalanine derivative and AFs have a tetrahydrocyclopenta[c]furo [3′,2′:4,5]furo[2,3-h]chromene skeleton. To supply to their low water solubility, mycotoxin solutions are prepared in organic solvents, such as acetonitrile [27,72] or methanol [56,58] or directly in neutral (pH 7.5) [42,67] and basic buffer (pH 8.5) [41].
- CE buffer: BGE, also named separation or electrophoretic buffer, represents a key issue in terms of buffer type, concentration, and (mainly) pH [36]. OTA is analyzed in neutral [56,58] or basic [27,41] BGE. The addition of βCD [56] can contribute to improving separation, exploiting its capacity to increase the analyte solubility and mobility, forming micellar-like structures. βCD proved to also be useful in the separation of ergot alkaloids [78]. For AFs, a basic buffer (borate) is the first choice BGE [67,72], with the addition of surfactants [67,72] and, eventually, organic solvents [67] to form hydrophobic/anionic interactions and maintain the analytes’ solubility, respectively [36].
- CE injection: A common hydrodynamic mode is used to inject the sample [36]. Often, prior to sample injection, a simple plug of methanol [27] or water-methanol [58] can be efficiently injected, contributing to a sample stacking and sensitivity improvement. Moreover, in aptamer affinity CE, a plug of desorbing buffer can be injected to a preconcentrate sampled, promoting analyte desorption from the aptamer [41].
- CE detection: The best detection system combined with CE remains LIF [27,41,42,44,67,72]. In particular, this detection is ideal for OTA and AFs, which have a native fluorescence. This allows us to reduce the sample preparation steps.
- Multi-analyte detection: This is a very critical point in the mycotoxin analysis topic. The recent work of Xiao et al. can clarify the issue, as can future directions for CE in the analysis of multi-mycotoxins [44], in which HPLC-MS [19] and HPLC-MS/MS are the tools of choice [15,19,25]. The approach of microchip-CE can be considered the proper strategy to obtain a rapid analysis that is useful for detecting contaminants, especially when a rapid check is required in food control quality. In particular, aptamer-based microchip-CE represents an interesting solution for simultaneous determination. The high specificity of aptamers for each different mycotoxin, together with the microchip-CE advantage, combining, in one device, the whole procedure from sample preparation to detection can allow a good resolution in a short amount of time. In addition, the integrated detection system (LIF) contributes to the rapidity and good sensitivity of the analysis.
- In non-microchip-CE, a sample pre-treatment (such as SPE) carried out with highly specific sorbent materials (MIPs or aptamers) could represent a future avenue, mainly in the simultaneous analysis of different mycotoxins, but also for mycotoxins belonging to the same family with similar chemical structures. Nowadays, this strategy, combined with CE techniques, remains unexplored, and MEKC is already a powerful method through which to separate different AFs (AFB1, AFB2, AFG1, and AFG2). In fact, the addition of a surfactant and a basic pH buffer can efficiently resolve the four AFs in 20 min [67] or even in 6 min [72]. The use of highly specific sorbents can be fundamental for those mycotoxins such as verrucosidin, which is a powerful neurotoxin [39]; it has a pyrone-type polyketide structure and it can represent a risk in food safety as it is associated with molds found in fermented meats [87]. Recently, new derivatives (penicyrone, norpenicyrone, methyl norpenicyrone, and methyl penicyrone) [88] or conformational isomers [89] were isolated, for example, from the marine fungus *Penicillium* sp. Y-50-10 and the possibility to use highly specific sorbents could solve the resolution issue.
- Sample matrices (liquid or solid) and sampling: CE techniques are well known to be ideal for aqueous samples/analytes. Notwithstanding this, non-aqueous samples can also be easily analyzed. Solid food has to be prepared in order to enable homogeneous mycotoxin contamination. In fact, homogenization, treatment with organic solvents, mixing, and centrifugation are the usual procedures reported in the literature and these ensure the resolution of this apparently critical point, as reported in the analysis of AFs [67,72], CIT [64], ergot alkaloids [78], and verrucosidin [39] in cereals, cheese, sausages, and ham slices.

## 5. Conclusions

For more than 50 years, mycotoxins have been well known as dangerous food contaminants present in trace amounts. The creation of very sensitive and rapid analytical methods became the key to detecting such analytes. The chromatographic approach represented the first-choice method, as recommended by the EC. Despite an evidently greater interest in the development of chromatographic rather than CE methods over the years, the study and investigation of pre-concentration approaches could make CE an effective alternative technique for routine analysis. Sample preparation, mainly with inline/online procedures, became a fundamental step to making CE competitive with GC or HPLC methods. The most studied and most promising technique is SPE, thanks to its wide range of support types, and as a consequence of its applications. CPE also deserves attention as, nowadays, it remains less explored, but it appears very promising in terms of its high sensitivity, together with its low cost and the environmental protection it provides. In fact, the study and exploration of sample preparation procedures could overcome the well-known CE limit, i.e., sensitivity.

Among CE modes, microchip-CE could represent the best choice in mycotoxin analysis, combining sample preparation, separation, and detection. These portable systems allow simple and rapid use, obtaining sensitivity values that are often even lower than those established by EC regulations. A promising tool for CE techniques and microchip-CE seems to be the use of aptamers, as their high specificity could allow simultaneous multi-mycotoxin analysis.

## Figures and Tables

**Table 1:** Types of mycotoxins, relative sample preparations and CE techniques.

Mycotoxin	Sample	Pre-Concentration Procedure	CE Mode	Refs
OTA	Wine	Offline SPE	CZE-UV	[56]
	River water	Offline/Inline SPE	CZE-UV	[58]
	Wine and beer	Inline aptamer-based μSPE Inline μIAC	CZE-LIF	[41]
	Model solute	Offline SPE	CZE–UV/LIF	[27]
AFs	Agricultural products	VALDS-ME	CZE-LIF	[72]
	(cereals)	-	-	-
	Rice	Sweeping	MEKC-LIF	[67]
AFB1	Corn flour	Aptamer probe addition	CZE-LIF	[42]
PAT	Infant apple juices	LLE	MEKC-UV	[69]
		LLE	CZE-UV	[70]
ZEA	Maize flour	SFE	CZE-AD	[74]
CIT	Red yeast rice and Monascus color	IAC	CZE-UV	[64]
Verrucosidin	Cheese, sausages, ham slices	TaqMan RTi-PCR	MEKC-UV	[39]
Ergot alkaloids	Cereals (grains and flours)	CPE	CZE-UV	[78]

**Table 2:** Types of mycotoxins analyzed by microchip-CE techniques.

Mycotoxin	Sample	Microchip	Immobilization	Detection	Refs
OTA	Green coffee extracts	Functionalized glass	Peptide-OTA conjugate covalently immobilized	CLD	[83]
	Foodstuffs (rice and corn)	Aptamer-based	/	LIF	[44]
	Corn	MIP	Ru(bpy)_3_^2+^	ECL	[84]
AFB1	Foodstuffs (rice and corn)	Aptamer-based	/	LIF	[44]
ZEA	Infant cereal milkshakes	Double-T glass	Magnetic beads	ECL	[81]

**Table 3:** Comparison between European Commission (EC) limits and sensitivity values obtained by CE methods reported in the present review for each mycotoxin and its relative food sample.

Mycotoxin	Sample	EC Maximum Levels [11]	CE-Methods Sensitivity Values
OTA	Wine	≤2 μg/Kg	LOD: 30 μg/L [56]
	Wine	≤2 μg/Kg	LOQ: 0.1 pg [41]
	Beer	not set	LOQ: 0.1 pg [41]
	Green coffee extracts	not set	LOQ: 7 μg/Kg [83]
	Foodstuffs (rice and corn)	≤5 μg/Kg	LOD: 0.021 μg/L [44]
AFs	Corn	≤5 μg/Kg	LOD: 0.03 μg/L [84]
	Agricultural products (cereals)	≤4 μg/Kg	LOQs: 0.007–0.300 μg/L [72]
	Rice	≤4 μg/Kg	LOQs: 0.13–1.74 μg/L [67]
AFB1	Corn flour	≤2 μg/Kg	LOQ: 0.156 μg/L (0.5 nM) [42]
	Foodstuffs (rice and corn)	≤2 μg/Kg	LOD: 0.026 μg/L [44]
PAT	Infant apple juices	≤10 μg/Kg	LOQ: 2.5 μg/L [69] LOQ: 17.9 μg/L [70]
ZEA	Maize flour	≤75 μg/Kg	LOD: 0.25 μg/L [74]
	Infant cereal milkshakes	≤20 μg/Kg	LOD: 0.4 μg/L [81]
CIT	Red yeast rice and Monascus color	≤100 μg/Kg	not present [64]
Verrucosidin	Cheese, sausages, ham slices	not set	LOD: 0.1 pg [39]
Ergot alkaloids	Cereals (grains and flours)	not set	LODs: 2.2–2.6 μg/Kg [78]
